# Atheroma of the Innominate Artery Presenting as a Transient Ischemic Attack

**DOI:** 10.7759/cureus.3961

**Published:** 2019-01-25

**Authors:** Moin Hassan, Ateeq Mubarik, Chirag Patel, Furqan Haq, Salman Muddassir

**Affiliations:** 1 Internal Medicine, North Shore Medical Center, Salem, USA; 2 Internal Medicine, Oak Hill Hospital, Brooksville, USA

**Keywords:** innominate artery atheroma, innominate artery atheroma anticoagulation, neuroprotective device, transient ischemic attack, innominate artery stenting, supra aortic atherosclerotic lesions

## Abstract

Supra-aortic atherosclerotic lesions, including innominate artery atheromas, are an uncommon but established cause of transient ischemic attacks, stroke, upper extremity ischemia, and vertebrobasilar insufficiency.

We present a patient with a transient ischemic attack admitted with right hemispheric symptoms who was found to have a severe ulcerated innominate artery atheroma. The patient underwent an aortic arch angiogram with stenting of the innominate artery.

The proper diagnosis, treatment, and management of innominate artery atheromas are imperative to prevent further cardiovascular morbidity and mortality in patients. Currently, both endovascular and surgical options are available for revascularization, and there have been no randomized controlled trials comparing endovascular versus open repair to standardize one as the standard of care over the other. No randomized controlled trials are examining the benefit of dual versus single antiplatelet therapy post-stenting in supra-aortic atherosclerotic lesions. We believe that this topic warrants further research and needs evidence-based guidelines to help direct physicians about treatment and management.

## Introduction

Supra-aortic atherosclerotic lesions, including innominate artery atheromas, play a major role in the development of ischemic lesions in the anterior and posterior cerebrovascular circulation and contribute to significant cardiovascular morbidity and mortality [1]. We present a patient who came in with right hemispheric symptoms and was found to have an innominate artery atheroma.

## Case presentation

**Figure 1:**
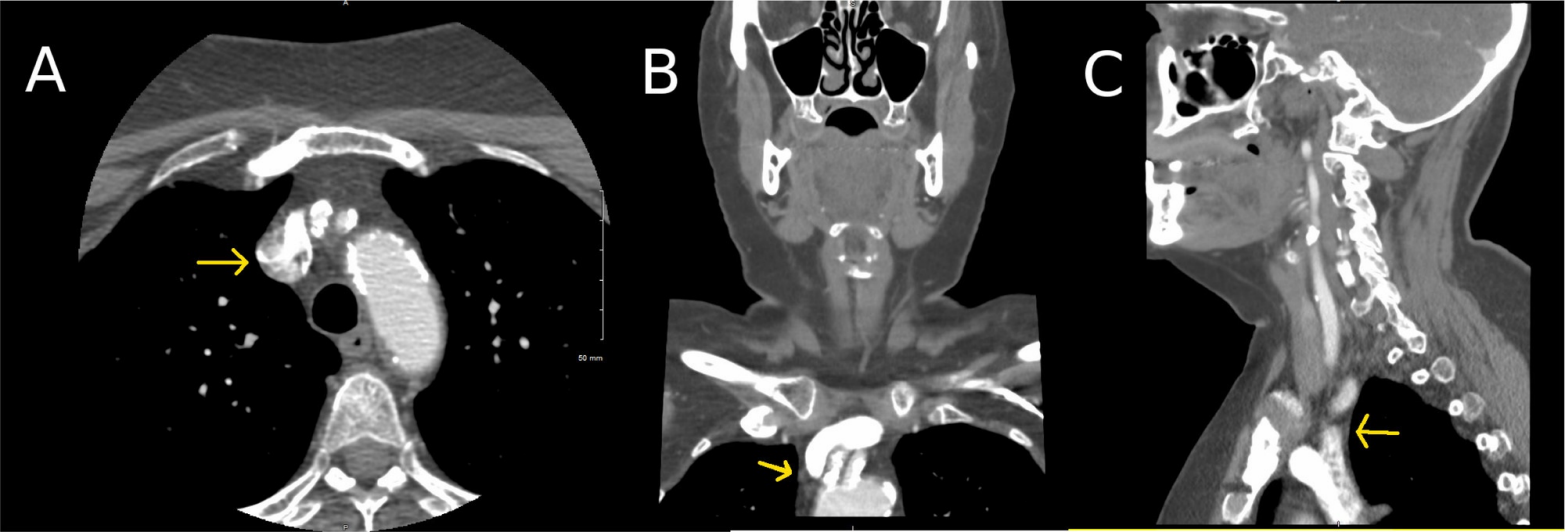
CTA of the neck showing 80% stenosis at the origin of the innominate artery (A) Axial View; (B) Coronal View; (C) Sagittal View CTA: Computed tomography angiography

A 69-year-old Caucasian female, with a past medical history significant for hypertension, came in with the chief complaint of unilateral numbness involving the left side of the face and both left upper and lower extremities, which lasted for about 10 to 15 minutes and resolved spontaneously. On examination, the National Institutes of Health Stroke Scale (NIHSS) score was 0, and the ABCD2 (A: age; B: blood pressure at presentation; C: clinical features; D: duration of symptoms) score was 0. The presence of diabetes (1 point) score was 3. There was carotid bruit bilaterally with higher intensity on the right and no focal neurological deficits. Stroke alert was called initially in the emergency department. A stat computed tomography (CT) brain revealed no acute intracranial abnormalities. The patient was not a candidate for alteplase, as the NIHSS is 0 and the symptoms had resolved, and aspirin 325 mg was given immediately. Magnetic resonance imaging (MRI) brain was done the same day and showed no acute ischemic or hemorrhagic infarct. We diagnosed the patient with a transient ischemic attack, and further workup was initiated to evaluate the underlying cause. The 12-lead electrocardiogram and cardiac monitoring showed no dysrhythmias, including atrial fibrillation. The transthoracic echocardiogram ruled out any intracardiac source of emboli. The carotid ultrasound revealed 50%-69% stenosis of the left internal carotid artery and no stenosis in the right carotid system. She had a CT angiogram of the neck and the great vessel and was found to have 80% stenosis at the origin of the innominate artery from the aortic arch, with no evidence of any significant stenosis in the right internal carotid artery (Figure 1).

There was about 50% stenosis at the origin of the left common carotid artery of the aortic arch, with mild stenosis of about 40% in the left internal carotid artery. Because of these findings, cardiology was consulted to evaluate the innominate artery stenosis, which recommended an angiogram and possible stent placement. We initially heparinized the patient for the procedure. The cardiologist placed a 6-French sheath into the right brachial artery after using local anesthesia and with the help of the ultrasound and micropuncture technique. Following this, a J-wire was used to enter into the aortic arch, but it would not advance, so a guidewire was placed and the innominate artery lesion crossed. Then, a marker pigtail catheter was set, and angiography of the arch and the proximal subclavian and brachial artery angiography was performed. The aortic arch angiogram showed the innominate artery originating from the ascending portion of the aortic arch, and shortly after the origin from the aorta, it had a severe, ulcerated, eccentric, irregular plaque estimated to be about 80%, calculated using the North American Symptomatic Carotid Endarterectomy (NASCET) criteria. The plaque was deemed to be the source of the embolization and transient ischemic attack. A 7-French 45 cm long sheath was advanced but was unable to cross the lesion. Therefore, the lesion was ballooned using a 6 x 20 mm long balloon and the 7-French sheath was advanced. Then, an 8.0 x 38 mm long atrium-covered stent was deployed to decrease the risk of future embolic events. Following this, a 9.0 x 40 mm long balloon was placed and a balloon dilatation performed with good angiographic results. No complications were encountered during the procedure. The patient was discharged on dual antiplatelet therapy with aspirin and clopidogrel for six weeks and statin therapy, and after that, on aspirin and statin therapy indefinitely. On her three-month follow-up, the patient reported no further episodes of numbness of the left side of her body. There was no postoperative imaging done, as the patients had no postoperative complications or recurrence of her symptoms.

## Discussion

Supra-aortic atherosclerotic lesions, including innominate artery atheromas, are an uncommon but established cause of transient ischemic attacks, stroke, upper extremity ischemia, and vertebrobasilar insufficiency [2].

Atherosclerosis is the most common pathology of the innominate artery. Other less common pathologies include aneurysmal degeneration, dissection, vasculitis, infection, and fibrosis. Stenosis greater than 75% of the vessel's diameter or thick, ulcerated plaque or thrombus within the arterial lumen is considered a severe lesion [2]. Our patient had a severe lesion, with an ulcerated, eccentric, irregular plaque estimated to be about 80%.

Innominate artery atheromas are uncommon and difficult to diagnose. They represent only 0.5% to 2% of all vascular lesions [3]. Transesophageal echocardiography (TEE), which is the procedure of choice for identifying aortic arch atheromas, does not readily visualize the innominate artery due to anatomical constraints. In a retrospective review of 520 MRI studies of the great vessels, only 10 patients had innominate artery atheromas, and none were visualized on TEE. This also underscores the importance of further diagnostic evaluation with either a gadolinium-enhanced MR angiography (MRA) or a CT angiography (CTA), as in our case when no source of embolization is found on echocardiography [4].

Best medical therapy is recommended in all patients with symptomatic supra-aortic atherosclerotic lesions, to reduce cardiovascular morbidity and mortality [5]. Best medical treatment (BMT) includes best pharmacological therapy, including antihypertensive, lipid-lowering, antithrombotic drugs, and optimal glucose control in people with diabetes. It also involves non-pharmacological measures, such as smoking cessation, healthy diet, weight loss, and regular physical exercise [6].

The treatment of supra-aortic atherosclerotic lesions has evolved over the past half-century. Shimizu et al. reported the first surgical repair of these lesions in 1951, but over the following years, this transthoracic approach was associated with a high mortality rate of around 22% [7]^.^ Extra-anatomic supra-aortic trunk reconstruction was introduced in 1967 by Diethrich et al., which decreased the mortality from 22% to 5.6 % [8]. In the 1980s, after the successful application of percutaneous transluminal angioplasty (PTA) to the coronary artery, renal artery, and peripheral artery atherosclerotic lesions [9], PTA was then applied to treat supra-aortic atherosclerotic lesions [10]. Stenting was then introduced to address the issue of long-term patency and reduce the need for reintervention. Over the years, PTA and stenting of the innominate artery lesions have been performed with relative safety and satisfactory mid-term success [11]. Currently, both endovascular and surgical options are available for revascularization, and there have been no randomized controlled trials comparing endovascular versus open repair to standardize one as the standard of care over the other. However, in recent years, PTA and stenting of the innominate artery have become the treatment of choice in most centers due to the reduced hospital stay and decreased complication and mortality rates, with open surgical repair only being indicated on unsuccessful endovascular treatment [3]. Recently, a “hybrid” approach by the surgical cut-down of the carotid artery and retrograde recanalization has been used. This approach allows neuroprotection (by clamping and flushing the carotid), and the lesion is easily crossed (increased pushability), with intermediate-term outcomes consistent with recent literature — however, there is no data comparing this technique with surgery alone or with endovascular treatment alone [12].

There are multiple approaches to access the innominate artery, with some authors preferring a retrograde brachial approach [13]while other authors prefer an antegrade femoral approach [11]. The proponents of the femoral approach give the following rationale for their preference: 1) As they do diagnostic angiography using the femoral approach and prefer that the procedure is completed in the same session and with the same access [11]; (2) The puncture site complication rate is lower for the femoral approach than for brachial puncture especially in cases when large sheaths are necessary [11]. The proponents of the brachial approach give the following rationale for their preference: (1) In anterograde access via a femoral artery, there is greater difficulty in using guidewires to cross steno-occlusive lesions; (2) less stability of the catheters and the sheaths carrying the stent with the femoral approach; and (3) increased risk of cerebral embolization in the presence of calcifications or atherothrombosis of the aortic arch [13]. Our cardiologist preferred the brachial access method and his rationale echoed with that of Moncalvo et al. [13]. His main reason for brachial access was that because of the retrograde approach to the lesion, there are fewer chances of cerebral embolization. There is currently no evidence on the risk of distal atheroembolization with the retrograde approach through the brachial artery versus the anterograde approach through the femoral artery. The benefits and risks of each method are physician-dependent and are based on their own clinical experience. There are no randomized controlled trials that show evidence of one approach being superior to another.

The use of neuroprotection devices in this procedure is controversial and technically difficult. The retrograde approach, the usage and positioning of the stent-graft for a soft plaque with high atherothrombotic embolic risk immediately after having crossed the lesion with the sheath, is hypothesized to decrease the risk of distal atheroembolization because it entrapped the plaque between the stent and the artery walls [13]. However, no evidence exists whether these measures decrease the risk of distal embolization. In all of the previous 13 cohort studies with supra-aortic atherosclerotic lesions and a total combined patient population of 616, neuroprotection devices were not used during the majority of the procedures [13]. Out of these 13 cohort studies, five studies reported procedural and post-procedural neurological complications. Paukovits et al. said 2.6% transient ischemic attacks in 77 patients [3], van Hattum at al. reported 4% transient ischemic attacks in 30 patients [14], Huttl et al. reported 2% left occipital infarctions and 6% transient ischemic attacks in 89 patients [11], Korner et al. reported 9% cerebrovascular thromboembolism in 43 patients [15], and Motarjeme et al. said 0.76% transient ischemic attacks in 131 patients [16].

Currently, no data exist regarding the benefit of dual antiplatelet versus single antiplatelet after supra-aortic stenting [17]. Most centers use clopidogrel (75 mg) and low-dose aspirin for one to three months post-stenting, which can be prolonged up to 12 months in some instances. Our patient received clopidogrel and baby aspirin for six weeks and then aspirin indefinitely.

## Conclusions

Innominate artery atheromas are an uncommon but important source of transient ischemic attacks and stroke. Their proper diagnosis, treatment, and management are imperative to prevent further cardiovascular morbidity and mortality in patients. We believe that this topic warrants further research and needs evidence-based guidelines to help direct physicians about treatment and management.
